# Ultra-Processed Foods Are Not “Real Food” but Really Affect Your Health

**DOI:** 10.3390/nu11081902

**Published:** 2019-08-15

**Authors:** Amelia Marti

**Affiliations:** 1 Department of Nutrition, Food Science and Physiology, University of Navarra, 31008 Pamplona, Spain; amarti@unav.es; Tel.: +34-948-425600; 2 IdiSNA (Navarra Institute for Health Research), 28903 Pamplona, Spain; 3 Center of Biomedical Research in Physiopathology of Obesity and Nutrition (CIBEROBN), Institute of Health Carlos III, 28029 Madrid, Spain

Poor eating habits, such as the increasing consumption of highly processed products, have deleterious effects on health status and represent a serious challenge for public health systems. They are not “real food” but formulations of food substances often modified by chemical processes and then assembled into ready-to-consume hyper-palatable food (cosmetic food) [1]. In this group, a large variety of industrially processed food products, such as savory snacks, reconstituted meat products, pre-prepared frozen dishes, and soft drinks among other food items, are included. Thus, it is very difficult to categorize them [2,3]. Three systems are reported to classify foods and beverages based on degree of industrial food processing [4]. The Nova system, developed in Brazil and used internationally in research, and two of them based on the U.S. diet: Specifically, a system developed by the International Food Information Council (IFIC) and used to examine the nutrient quality of foods consumed by Americans by processing category, and another created by researchers at the University of North Carolina at Chapel Hill (UNC) that categorizes all barcoded foods items sold in U.S. supermarkets.

In a recent paper, Bleiweiss-Sande et al. [4] evaluated the robustness of food processing classification systems (Nova, IFIC, and UNC). They also assessed their utility as a measure of healthfulness in children’s diets, using data on the top 100 most commonly consumed foods by children. They indicated that there are several differences; for example, while the Nova system divides foods into four categories, IFIC splits them into five categories, and UNC into seven categories of processing. They observed that, as expected, the UNC and Nova systems had the highest agreement, since the UNC system was developed based on the Nova framework. Interestingly, they found considerable overlap between foods classified as moderately processed with minimally and highly processed foods, but nutrient concentrations were not strong predictors of processing category in any of the three systems. They claim that there is a need for a commonly accepted classification system and definitions to describe processing categories and that the new framework should consider food categorizations that aligned with nutrient content to increase usefulness.

From a public health view, it is important to choose the best tool to measure consumption of ultra-processed food (25 and 60% of total daily energy intake) in order to examine their influence on the onset of obesity and non-communicable diseases, such as cardiovascular diseases, diabetes, respiratory diseases, or cancer [5,6,7]. Rauber et al. [8] evaluated the consumption of ultra-processed foods using the Nova system in 9374 (4738 adults and 4636 children) U.K. participants. They also examined their potential association with nutrients known to affect the risk of chronic non-communicable diseases. Subjects had an average energy intake of 1764 kcal/day, with 30.1% of calories coming from unprocessed or minimally processed foods, 4.2% from culinary ingredients, 8.8% from processed foods, and 56.8% from ultra-processed foods. These were mainly: industrialized packaged breads, packaged pre-prepared meals, breakfast cereals, reconstituted meat products, confectionery, biscuits, pastries, buns and cakes, industrial chips (French fries), and soft and fruit drinks/juices. This high dietary share of ultra-processed foods in the United Kingdom was similar to data from the United States and Canada, but greater than values reported in France or Brazil [8].

Recently, prospective studies conducted in children indicated that consumption of ultra-processed foods (following the Nova system) was associated with added sugar content in the diets or even influenced anthropometric and glucose profile [9,10]. Notably, two large European cohort studies reported positive associations between consumption of ultra-processed foods (following the Nova system) and cardiovascular disease and all-cause mortality in adults [5]. First, Srour et al. [11] showed an association between an absolute 10% increase in dietary ultra-processed food and significantly higher rates of overall cardiovascular disease, coronary heart disease, and cerebrovascular disease. Secondly, Rico-Campa et al. [12] found that a higher consumption of ultra-processed foods (>4 servings daily) was independently associated with a 62% relatively increased hazard for all-cause mortality in 19,899 participants of the SUN study. Similar findings were also reported in the U.S. population (NHANES III, 1988–1994) using the NOVA system [7]. Individuals followed for 19 years had a 31% higher risk of all-cause mortality when they were in the highest quartile of frequency of ultra-processed food intake (e.g., sugar-sweetened or artificially sweetened beverages, sweetened milk, sausage or other reconstructed meats, sweetened cereals, confectionery, and desserts).

Although these results need to be validated in other populations and study designs, a number of public health authorities worldwide have advised limiting the consumption of ultra-processed foods [11]. Currently, there is a need for a commonly accepted classification system to describe processing categories that best reflect the nutrient content to increase applicability in the public health arena.
